# Self-Compassion Demonstrating a Dual Relationship with Pain Dependent on High-Frequency Heart Rate Variability

**DOI:** 10.1155/2020/3126036

**Published:** 2020-02-18

**Authors:** Shuxiang Tian, Xi Luo, Xianwei Che, Guizhi Xu

**Affiliations:** ^1^State Key Laboratory of Reliability and Intelligence of Electrical Equipment, Hebei University of Technology, Tianjin, China; ^2^Key Laboratory of Electromagnetic Field and Electrical Apparatus Reliability of Hebei Province, School of Electrical Engineering, Hebei University of Technology, 8 Guangrong Road, Hongqiao District, Tianjin 300130, China; ^3^College of Preschool and Primary Education, China West Normal University, Nanchong, China; ^4^Center for Cognition and Brain Disorders, Institutes of Psychological Sciences, Hangzhou Normal University, Hangzhou, China; ^5^Zhejiang Key Laboratory for Research in Assessment of Cognitive Impairments, Hangzhou, China

## Abstract

One previous study indicated the significance of trait self-compassion in psychological well-being and adjustment in people with chronic pain. Higher-frequency heart rate variability (HF-HRV) was found to be closely associated with self-compassion and pain coping. The current study was therefore designed to investigate the relationship between self-compassion and experimental pain as well as the impact of HF-HRV. Sixty healthy participants provided self-reported self-compassion and underwent a cold pain protocol during which HF-HRV was evaluated. Results demonstrated a dual relationship between self-compassion and pain, dependent on the level of HF-HRV during pain exposure. Specifically, self-compassion was associated with lower pain in the condition of higher HF-HRV, while there was an inverse relationship between self-compassion and pain when HF-HRV was lower. Our data indicate the significance of HF-HRV in moderating the association between self-compassion and experimental pain.

## 1. Introduction

Self-compassion generally entails the capability to be kind and caring toward oneself in times of suffering, failure, or perceived inadequacy [1]. A large number of studies have established the protective influence of self-compassion on psychosocial distress, including social anxiety [2, 3], burnout [4], and trauma [5]. Beyond this evidence, recent studies have indicated the significance of self-compassion in pain experience [6, 7]. One study found that a greater ability to show self-compassion was associated with a lower negative effect and catastrophizing in people with chronic pain [6].

However, the number of studies to support the benefits of self-compassion in pain is highly limited. More studies are therefore required to improve our understanding on the role of self-compassion in pain experience. Moreover, the literature has indicated a close relationship between self-compassion and heart rate variability (HRV) as well as its role in pain coping. Self-compassion was found to be associated with increased HRV in the context of a stress, in which HRV, especially high-frequency HRV (HF-HRV), was thought to reflect parasympathetic activity and the regulatory control over sympathetic arousal [8]. In contrast, recent evidence demonstrated that pain could suppress HRV [9]. These studies therefore indicate the potential of increased HRV in linking self-compassion with pain reduction.

The current study was designed to investigate the relationship between self-compassion and pain as well as the impact of HF-HRV on this association. Healthy participants provided self-reported self-compassion and then underwent a cold pain protocol. Mediation and moderation models were considered. In the case of mediation, we hypothesize that self-compassion is associated with higher HF-HRV, which in turn reduces pain experience. Meanwhile, in the case of moderation, self-compassion is assumed to be associated with lower pain in the condition of higher level of HF-HRV. Both of the two cases would implicate the role of HF-HRV in the relationship between self-compassion and pain and possibly provide insight into the therapeutic role of self-compassion in chronic pain.

## 2. Materials and Methods

### 2.1. Participants

Sixty healthy, pain-free, right-handed adults participated in this study. In order to reduce expectancy effects, participants were told that the aim of the study is to examine heart rhythm to cold water. ECG data from three participants were contaminated, and data from 57 participants were therefore analysed (27 males and 30 females, age range: 19–33 years, Mean = 20.28, SD = 2.38). Exclusion criteria included use of psychoactive medication or a history or current diagnosis of a psychiatric disorder, as assessed by the Mini International Neuropsychiatric Interview (MINI) [10]. All study participants provided informed consent, and the study was approved by the Ethics Committee in the China West Normal University. This study was conducted in accordance with the Declaration of Helsinki.

### 2.2. Experimental Design and Procedure

Participants recruited to this study underwent a single-session design protocol. Following consent, participants were asked to fulfill the Self-Compassion Scale (see below *Self-Compassion Scale*). Participants were then set up with the ECG recording system, which was followed by a 3-minute cold pain exposure (see below *Pain Stimulation*).

### 2.3. Self-Compassion Scale (SCS)

The 26-item Self-Compassion Scale was used to measure individual differences in self-compassion [11]. It is composed of six subscales: self-kindness, self-judgment, common humanity, isolation, mindfulness, and overidentification. The total score was created by calculating the grand mean score of subscales after reversing the coding responses to the negatively worded items. Participants were asked to indicate how they typically act toward themselves in difficult times using a five-point Likert scale (from 1 “never” to 5 “almost always”). SCS showed well-established psychometric characteristics with an internal consistency of 0.92 [11]. Chen et al. [12] reported Cronbach's alpha (0.83) and test-retest reliability (0.89) of the Chinese version.

### 2.4. ECG Recording

A BITalino (r) evolution Board Kit BT (BITalino, Portugal) was used to record ECG (http://bitalino.com/en/). Three Ag/AgCl electrodes were used, with two electrodes being attached to the bilateral clavicle area within the rib cage, respectively, and one electrode to the lower edge of left rib cage. Data were recorded using OpenSignals (r)evolution software (v.2017, BITalino, Portugal) at a sampling rate of 1,000 Hz.

### 2.5. Experimental Protocol

Participants underwent a 3-minute cold pain protocol which was divided into six consecutive 30 second blocks. In a 30 second block, participants viewed a fixation cross for 25 seconds and then rated “pain intensity at the moment” on a scale of 0–10 (0 = no pain; 10 = worst pain imaginable) in 5 seconds (PowerPoint, Microsoft Corporation). In order to avoid socially desirable behaviour [13], participants wrote the pain ratings on a piece of paper that could not be seen by the experimenter.

### 2.6. Pain Stimulation

A recent study demonstrated that an iced bottle can induce ongoing cold pain [14]. In the current study, participants were asked to hold a 0.5 L plastic bottle with iced water (−1°C) for 3 minutes. This protocol was used in a previous study by our group [15]. Participants were told to put the volar surface of the nondominant hand on the surface of the bottle and not to squeeze or avoid it, to minimize the variability of touching. The nondominant hand was selected according to the pain literature [16]. A fresh iced bottle was used for each participant for consistency.

### 2.7. Data Analysis

**Figure 1:**
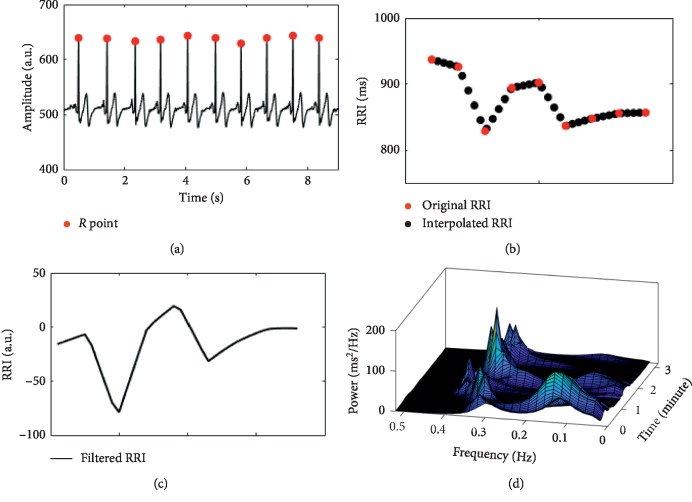
Analysis procedure of HF-HRV. For detailed information, please refer to the Methods section.

ECG data during pain exposure were analysed as illustrated in Figure 1. The Pan–Tompkins algorithm was used to identify the *R* points from the QRS complex (Figure 1(a)) [17]. Artefacts were visually checked and edited according to the published guidelines [18]. The original R-R Intervals (RRIs) were calculated and then linearly interpolated to 4 Hz to obtain evenly sampled signals (Figure 1(b)) [19, 20]. In order to remove the slow drift, interpolated RRI waves were high-pass filtered with the cutoff frequency of 0.02 Hz [19] (Figure 1(c)). Filtered RRI waves were then used to calculate HRV using the time-varying autoregressive (TVAR) model which can capture the dynamics of HRV [21] (Figure 1(d)). In particular, the TVAR model is suggested to be able to provide accurate estimation of the power spectrum [22], and it has been used in the investigation of beat-to-beat spectra during ongoing pain [19]. The model order was set to 12 according to the literature [22]. HF-HRV was expressed as the relative value of high-frequency component (0.15–0.4 Hz) in proportion to the total power minus the very low-frequency component (0–0.04 Hz) [23]. The relative values of HF-HRV are suggested to emphasize the controlled and balanced behaviour of the sympathetic and parasympathetic branch of the autonomic nervous system (ANS) [23].

### 2.8. Statistical Analyses

Correlation analyses were initially conducted among self-compassion, HF-HRV, and pain using SPSS (version 23; IBM Corp, Armonk, NY). The area under the curve (AUC) of pain and HF-HRV during the 3-minute pain was calculated using the linear trapezoidal rule. The AUC approach was employed as it provides a summary measure of pain or HF-HRV dynamics across a specified time window. A mediation model was not performed as there was no significant association between self-compassion and pain. A moderation analysis was conducted using PROCESS with the bootstrapping method [24]. Specifically, the model was set to “1” (i.e., conditional effect), and self-compassion, HF-HRV, and pain were specified as the independent, moderator, and dependent variable, respectively. The bias-corrected and accelerated (BCa) bootstrap estimates were based on 5,000 bootstrap samples. As this was a cross-sectional design, in a supplementary analysis, we exchanged self-compassion, HF-HRV, and pain within a moderation model.

## 3. Results

### 3.1. Descriptive and Correlational Analysis

**Figure 2:**
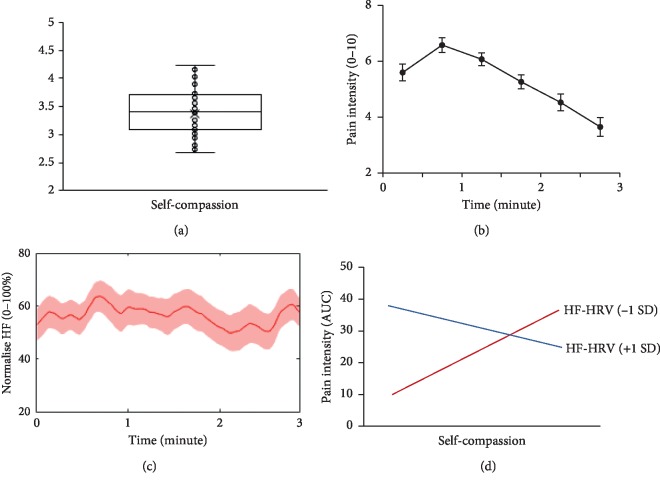
Self-compassion, pain, and the moderating influence of HF-HRV. (a) The boxplot of self-compassion. (b) The dynamics of pain intensity ratings (mean ± SEM). (c) The frequency-based HRV. The shaded area represents the SEM. (d) HF-HRV moderated the association between self-compassion and pain. Self-compassion was associated with more pain when HF-HRV was lower (−1 SD), while related to lower pain in individuals with higher HF-HRV (+1 SD). a.u. denotes arbitrary unit. AUC denotes the area under the curve. SEM denotes the standard error of the mean.

Participants reported a total self-compassion score of 3.38 (SD = 0.44) (Figure 2(a)).  Figure 2(b) demonstrates the dynamics in pain ratings. A one-way ANOVA on pain ratings indicated that pain kept increasing by the end of the first minute (Time 2 vs. Time 1, *P*_Bonf_=0.001), remained high in the second minute (Time 4 vs. Time 1, *P*_Bonf_ > 0.05), and then decreased by the end of pain exposure (Time 6 vs. Time 1, *P*_Bonf_=0.002). Correlational analyses found no significant association among self-compassion, HF-HRV, or pain (*P*_*s*_ > 0.05).

### 3.2. Moderation Analysis

Figure 2(c) demonstrates the HF-HRV dynamics across the pain exposure. HF-HRV was found to moderate the relationship between self-compassion and pain ratings (Δ*R*^2^ = 0.15, *F*_1_,_53_ = 9.69, *P*=0.003). The moderation analysis further revealed that self-compassion was associated with increased pain (*P*=0.019) when HF-HRV was lower (≤−1 SD), while self-compassion was associated with lower pain (*P*=0.046) when HF-HRV was higher (≥1 SD) (Figure 2(d)). The supplementary analysis revealed no other significant models (all *P*_*s*_ > 0.05).

## 4. Discussion

The current study was designed to investigate the association between trait self-compassion and experimentally induced pain as well as the role of HF-HRV in this relationship. Our results demonstrated a dual relationship between self-compassion and pain, dependent on the level of HF-HRV during pain administration. Self-compassion was associated with lower pain when HF-HRV was relatively high. Meanwhile, self-compassion was inversely related to pain experience in individuals with lower HF-HRV. Our data indicate the particular importance of HF-HRV in moderating the relationship between self-compassion and pain.

Our data demonstrated a moderation effect of HF-HRV in the association between self-compassion and pain experience. One previous study found that, in people with chronic pain, trait self-compassion was associated with lower negative affect and higher ability to be compassionate (i.e., lower catastrophizing and rumination) using an attribution protocol [6]. In another study, self-compassion was a significant predictor of lower level of pain catastrophizing and pain disability among patients who have persistent pain and who are obese [25]. In line with these findings, our data demonstrate the relationship between self-compassion and pain experience dependent on the level of HF-HRV.

Findings in the current study indicated that self-compassion was associated with pain experience dependent on the level of HF-HRV. More interestingly, our results indicated a double dissociation between self-compassion and pain (Figure 2(d)). Self-compassion means to treat oneself with kindness, acceptance, and a sense of common humanity in times of suffering [1]. Our findings highlight the importance of HF-HRV in moderating the association between self-compassion and pain. Pain serves to protect the body whereby the energy resources are allocated by the autonomic nervous system (ANS) [26]. Pain can activate the sympathetic branch while suppressing the parasympathetic branch of the ANS [9]. Meanwhile, HF-HRV is believed to be closely and strongly associated with cardiac vagal tone (i.e., parasympathetic tone) which reflects the regulatory control over sympathetic arousal [9]. Therefore, our findings may be more related to the role of HF-HRV in the regulation of pain-related arousal. Similarly, recent studies showed that increased HF-HRV was associated with decreased pain experience in a mindfulness meditation or simply a compassionate self-talk protocol [27, 28]. Overall, we present interesting findings suggesting the particular significance of HF-HRV in moderating the relationship between self-compassion and pain experience.

It is noted that our data did not support the role of HF-HRV in mediating the influence of self-compassion on pain. A moderation model is different from a mediation one, with the latter being able to provide more information on the causal relationship between variables [29]. Nonetheless, our findings support the role of HF-HRV in linking self-compassion with pain experience.

There are other potential approaches to investigate the association between self-compassion and pain experience beyond HRV. Electroencephalogram (EEG) and functional imaging studies have tried to reveal the mechanisms of nociceptive transmission. EEG evidence has suggested that pain may suppress alpha activity but increase gamma activity which underlies the nociceptive transmission and integration, respectively [30]. In addition, pain is suggested to be mediated by a “spinothalamocortical” pathway [31]. Findings from these imaging methods would enrich our understanding on the role of self-compassion in pain experience. Moreover, the literature has indicated a close relationship between self-compassion and coping strategies (e.g., emotion regulation and cognitive reconstructing) as well as their impact on health outcomes [32]. Future studies may wish to investigate the role of coping strategies in the association between self-compassion and pain.

We acknowledge some limitations in the current study. We recruited an easy sample with a relatively narrow age range, which limits the conclusions to be generalised to other age ranges, such as old adults. Indeed, age plays a role in both self-reported self-compassion [33] and pain experience [34]. Findings in this study therefore need to be further examined in other age groups. Other specific physical conditions or medications that could have influenced pain or HRV were not considered. Nonetheless, the participants were screened using MINI [10] and were free of pain. We presented results from healthy participants in the current study, which warrants further investigation in people with chronic pain. Purdie and Morley [6] demonstrated the importance of self-compassion in psychological well-being and adjustment in people with chronic pain. Future studies could further examine the relationship between self-compassion and pain experience in chronic pain populations as well as the moderating impact of HF-HRV. Although a short-term HRV measurement was used in this study, long-term recordings and HRV measurements need to be considered [23]. Other variables that potentially influence HRV measurements (e.g., core body temperature and circadian rhythm) were not controlled. In addition, we used an iced bottle [14] to induce cold pain instead of a cold pressure test [35]. Condensation is expected on the surface of the bottle. However, this is not expected to affect the results as we have carefully controlled the timing to bring out the bottle from the freezer.

To conclude, HF-HRV moderates the relationship between self-compassion and pain experience. Trait self-compassion has a dual association with experimentally induced pain dependent on the level of HF-HRV during pain administration. These findings may have implications for pain management. Changing the mindset in a more compassionate fashion toward oneself may be effective in pain coping. Moreover, our data provide empirical evidence for the development of compassionate interventions in the management of chronic pain [25, 36].

## Data Availability

The data used to support the findings of this study are available from the corresponding author upon request.

## Supplementary Materials

Supplementary Materials STROBE statement: checklist of items that should be included in reports of observational studies.
