# Corrigendum to “Self-Compassion Demonstrating a Dual Relationship with Pain Dependent on High-Frequency Heart Rate Variability”

**DOI:** 10.1155/2021/6596375

**Published:** 2021-02-15

**Authors:** Shuxiang Tian, Xi Luo, Xianwei Che, Guizhi Xu

**Affiliations:** ^1^State Key Laboratory of Reliability and Intelligence of Electrical Equipment, Hebei University of Technology, Tianjin, China; ^2^Key Laboratory of Electromagnetic Field and Electrical Apparatus Reliability of Hebei Province, School of Electrical Engineering, Hebei University of Technology, 8 Guangrong Road,Hongqiao District, Tianjin 300130, China; ^3^College of Preschool and Primary Education, China West Normal University, Nanchong, China; ^4^Center for Cognition and Brain Disorders, Institutes of Psychological Sciences, Hangzhou Normal University, Hangzhou, China; ^5^Zhejiang Key Laboratory for Research in Assessment of Cognitive Impairments, Hangzhou, China

In the article titled “Self-Compassion Demonstrating a Dual Relationship with Pain Dependent on High-Frequency Heart Rate Variability” [1], the authors would like to clarify that Figure 1 was derived from a previous publication by the authors in [2]. Permission was obtained from Elsevier for the publication of this figure, and the figure legend should be corrected as follows, including the additional reference [37] [2]:

“Figure 1: Analysis procedure of HF-HRV. For detailed information, please refer to the Methods section (derived from [37] which has been reproduced with permission from Elsevier).”
